# The Mould War: Developing an Armamentarium against Fungal Pathogens Utilising Thymoquinone, Ocimene, and Miramistin within Bacterial Cellulose Matrices

**DOI:** 10.3390/ma14102654

**Published:** 2021-05-18

**Authors:** Sam Swingler, Abhishek Gupta, Hazel Gibson, Wayne Heaselgrave, Marek Kowalczuk, Grazyna Adamus, Iza Radecka

**Affiliations:** 1 Wolverhampton School of Sciences, Faculty of Science and Engineering, University of Wolverhampton, Wulfruna Street, Wolverhampton WV1 1LY, UK; h.gibson@wlv.ac.uk; 2 Research Institute in Healthcare Science, Faculty of Science and Engineering, University of Wolverhampton, Wulfruna Street, Wolverhampton WV1 1LY, UK; a.gupta@wlv.ac.uk (A.G.); w.heaselgrave@wlv.ac.uk (W.H.); 3 Institute of Health, Faculty of Education, Health and Wellbeing, University of Wolverhampton, Jerome K Jerome Building, Gorway Road, Walsall Campus, Walsall WS1 3BD, UK; 4 Department of Biomedical Science, University of Wolverhampton, MA Building, Wulfruna Street, Wolverhampton WV1 1LY, UK; 5 Centre of Polymer and Carbon Materials, Polish Academy of Sciences, M. Curie-Sklodowskiej 34, 41-819 Zabrze, Poland; marek.kowalczuk@cmpw-pan.edu.pl (M.K.); gadamus@cmpw-pan.edu.pl (G.A.)

**Keywords:** antifungal, thymoquinone, ocimene, miramistin amphotericin b, bacterial cellulose, wound dressing

## Abstract

An increase in antifungal resistance has seen a surge in fungal wound infections in patients who are immunocompromised resulting from chemotherapy, disease, and burns. Human pathogenic fungi are increasingly becoming resistant to a sparse repertoire of existing antifungal drugs, which has given rise to the need to develop novel treatments for potentially lethal infections. Bacterial cellulose (BC) produced by *Gluconacetobacter xylinus* has been shown to possess many properties that make it innately useful as a next-generation biopolymer to be utilised as a wound dressing. The current study demonstrates the creation of a pharmacologically active wound dressing by loading antifungal agents into a biopolymer hydrogel to produce a novel wound dressing. Amphotericin B is known to be highly hepatotoxic, which reduces its appeal as an antifungal drug, especially in patients who are immunocompromised. This, coupled with an increase in antifungal resistance, has seen a surge in fungal wound infections in patients who are immunodeficient due to chemotherapy, disease, or injury. Antifungal activity was conducted via Clinical & Laboratory Standards Institute (CLSI) M27, M38, M44, and M51 against *Candida auris*, *Candida albicans*, *Aspergillus fumigatus,* and *Aspergillus niger*. This study showed that thymoquinone has a comparable antifungal activity to amphotericin B with mean zones of inhibition of 21.425 ± 0.925 mm and 22.53 ± 0.969 mm, respectively. However, the mean survival rate of HEp-2 cells when treated with 50 mg/L amphotericin B was 29.25 ± 0.854% compared to 71.25 ± 1.797% when treated with 50 mg/L thymoquinone. Following cytotoxicity assays against HEp-2 cells, thymoquinone showed a 71.25 ± 3.594% cell survival, whereas amphotericin B had a mean cell survival rate of 29.25 ± 1.708%. The purpose of this study was to compare the efficacy of thymoquinone, ocimene, and miramistin against amphotericin B in the application of novel antifungal dressings.

## 1. Introduction

Wounds from various sources that have been in a prolonged state of irritation and rubor, with a high degree of exudate, are decidedly prone to becoming infected by various opportunistic and commensal organisms. The typical approach in treating these wounds is to reduce infection and increase more favourable conditions at the wound site for healing, thus encouraging a more suitable environment for successful re-epithelisation and angiogenesis [1,2].

Immunodeficiency arising from burn injuries poses a genuine and present threat to the successful healing of patients. The central difficulty in burn patients is the burn wound itself. Although the body as a whole reacts to a burn dependent on severity, thickness, and coverage, those with thermal injuries that are extensive typify a whole-body reaction. As a result of this reaction, the inflammation arising from the injury mandates that the wound is protected from infection and that there is a timely closure of the exposed areas. Burn wound infections and mortality rates are drastically decreased with the application of topical treatments such as silver sulfadiazine and mafenide acetate by nearly 50% [3,4].

Antifungal resistance is an ever-growing issue within the healthcare sector, seeing over 1.5 million people die annually from fungal infections [5,6]. Immunocompromised patients make up the bulk of these deaths, and with classical antifungal drugs such as the azoles becoming less effective in the treatment of infections as a result of decreased efficacy, this figure is most likely going to increase.

A dramatic increase in fungal infections in recent years is associated with several factors such as prolonged dosage of broad-spectrum antibiotics, immunosuppressive therapy, extended stays in hospitals, burns, and recently undergone surgery as well as malignancy [7,8].

*Candida* species are polymorphic yeasts that can exist in various morphological forms that facilitate survival under extreme microenvironments by forming biofilms or invading and destructing target tissues, primarily in the pseudo-hyphae and true hyphae morphological state [9]. Unlike other pathogenic organisms, morphogenic plasticity helps *Candida* species evade host immune responses and confers differential responses towards antifungal agents [10]. Being eukaryotic organisms, fungal specific drug targets are very few and thus, limited numbers of antifungal agents such as azoles, polyenes, allylamines, and echinocandins, et cetera are available on the market [11].

**Figure 1:**
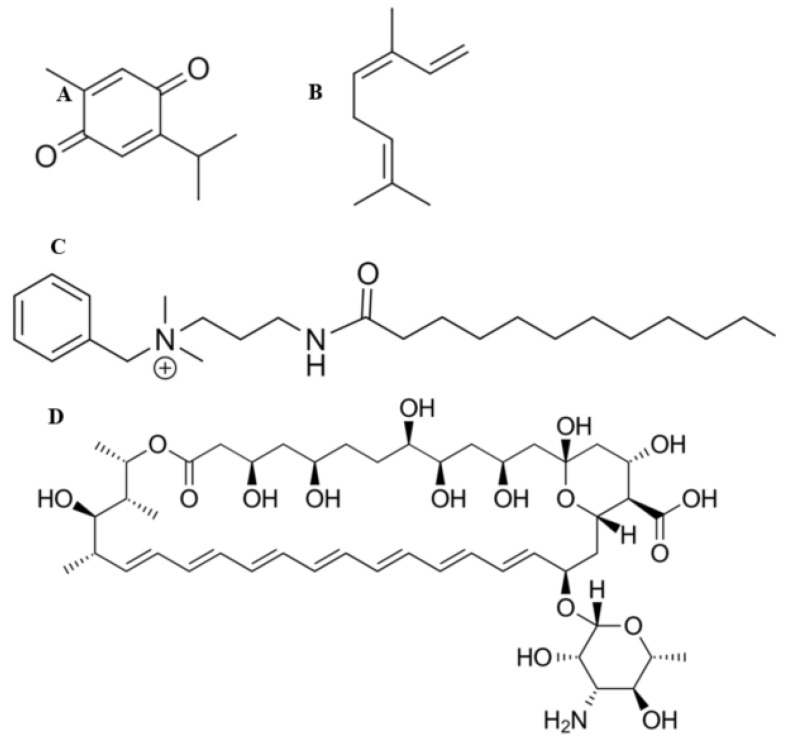
Chemical structures of thymoquinone (**A**), ocimene (**B**), miramistin (**C**), and amphotericin B (**D**).

Thymoquinone (Figure 1A) and ocimene (Figure 1B) are the major constituents of *Nigella sativa* and *Azadirachta indica*, respectively. It has been shown that both of these compounds possess various pharmacological qualities, which include antibacterial, anti-parasitic, anti-inflammatory, anti-viral, and antifungal properties [12,13,14]. They have also been shown to provide a level of protection against nephrotoxicity [15]. Miramistin (benzyl dimethyl [3-(myristoilamino) propyl] ammonium chloride monohydrate) (Figure 1C) is a topical antiseptic that was developed in the Soviet Union during the Cold War within the framework of the ‘Space Biotechnology Program’ [16].

Amphotericin B (Figure 1D) is the principal antifungal polyene agent with a broad-spectrum activity used to treat burn patients. The mode of the action revolves around the drug destabilising ergosterol within the cell wall of the fungi, thus causing rapid ion leakage culminating in cellular death [17]. The administration of amphotericin B ranges from 0.6 to 1 mg/kg/day and poses significant intolerance in burn patients who exhibit side effects such as hypoxia, hypertension, fevers, and more significantly, nephrotoxicity—although the introduction of liposomal formulations has eased this side effect [18]. This side effect limits the overall effectiveness of intravenously administering amphotericin B as tubular wasting is significantly reduced. Therefore, a method for delivering this drug to patients’ burn site is greatly needed [19]. The topical administration of amphotericin B to burn patients’ wounds would allow for rapid, localised control and prevention of fungal pathogens becoming disseminated, consequently minimising the risk of fungal infections becoming deep-rooted, which drastically increases the risk of death.

**Figure 2:**
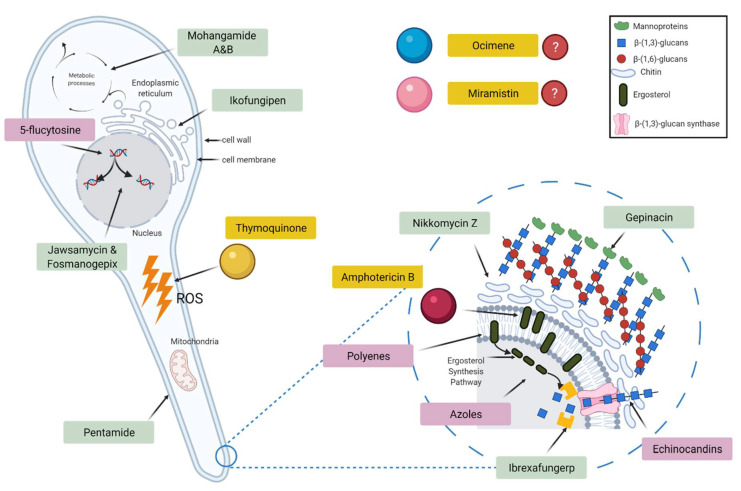
Schematic diagram showing the target sites of currently used antifungal drugs (pink), pipeline antifungals (green), and the test drugs used in this study (yellow).

Although the overall mechanism of the antifungal actions of both thymoquinone and ocimene is not fully understood, it is well documented that both thymoquinone and ocimene are sources of reactive oxygen species (ROS), leading to oxidative stress, which could play a significant role in the fungicidal properties [20,21]. However, the antifungal activity of miramistin is poorly documented (Figure 2).

The two most prevalent fungal genera responsible for burn wound infections are *Candida* and *Aspergillus* species. In the present study, *C. albicans, C. auris, A. fumigatus*, and *A. niger* are being investigated providing representative pathogenic organisms of both non-dermatophyte fungi and yeasts. *Candida* species are responsible for approximately 80% of nosocomial yeast infections, of which 4–11% of burn patients are diagnosed with invasive candidemia, which results in a 30–58% mortality rate [22]. However, *Aspergillus* species account for 0.3–7% of fungal wound infections in burn patients, resulting in invasive aspergillosis. Nevertheless, the highly angio-invasive nature of aspergillosis results in a 50–78% mortality rate [23,24,25].

Established dressings such as gauze and tulle form a barrier between the wound site and the external environment that keeps the wound site dry. However, they are unable to impart any anti-microbial activity directly or influence the wound-healing process [26]. Conversely, moist wound dressings such as bacterial cellulose dressings act as a barrier to infection and also maintain moisture levels around the wound. One advantage of these dressings is that they are easily removed from the wound site, thus avoiding further trauma during dressing changes. These dressings also respond to variations in moisture levels, thereby facilitating re-epithelialisation of the wound site [27]. In addition to absorbing and retaining excess wound exudate, hydrocolloid dressings also provide a cooling, soothing effect, which also reduces rubor and the sensation of pain [28].

Bacterial cellulose (BC) can be used as part of a biocompatible system; it is non-pyrogenic and hydrophilic, which make it innately suitable for wound treatment applications [29]. In addition, BC also contours superbly to the undulating surface of the skin, providing a uniform covering even in areas that are usually difficult to dress, such as the groin and neck. BC also provides protection to healing wounds due to the thickness of the dressing, which reduces further injury from further trauma. This protection further aids in the promotion of angiogenesis and rapid wound healing [30,31].

Bacterial cellulose is biosynthesised through the conjugation of linear homopolysaccharides and β-D-glucose units which are linked by 1,4-β-glycosidic linkages [1]. Once the exopolysaccharides have formed, they then randomly become organised into chains consisting of 10 to 15 individual chains of cellulose, resulting in cellulose nanofibers. These single chains of nanofibers become further entangled to form microfibrils, which are up to 100 times smaller than commercially available vegetal analogues [32,33,34].

Then, the synthesised cellulose microfibrils form ribbon bundles, which are 3–4 nm thick and 70–80 nm wide, which are what form the cellulose pellicle. This is achieved through inter and extra-chemical bonding, primarily hydrogen bonding between hydroxyl groups within between the cellulose fibres [35].

Due to the high levels of hydrogen bonding between the cellulose fibres, pores are formed within the cellulose, which possess an overall negative ionic charge resulting from hydroxyl groups and permit additional compounds to be embedded. This intrinsic physical property of cellulose allows for an increased level of influence over the bioactivity of the material as additional compounds, providing there is some level of positive ionic charge owing to the negative ions charge in the bacterial cellulose arising from the hydroxyl groups, such as antifungal agents, which can be easily incorporated [36].

As bacterial cellulose is a biopolymer, there are naturally varying degrees of pore size and distribution. These pores can be further influenced by the method of fermentation itself, whether it be static or agitated. Previous research conducted by Revin et al. (2019) showed that under agitated conditions, the bacterial cellulose forms spheres with a pore size of 165–330 μm, while under static conditions, the cellulose formed as a pellicle with an average pore size of 4 nm to 1000 µm in diameter [37,38].

The overall distribution and size of the pores within the bacterial cellulose pellicle greatly impacts both the physical and mechanical properties of the biomaterial, in that larger pores introduce greater voids resulting in a less dense material. Subsequently, these larger pores allow for larger molecules or substances to be loaded more easily as a result of increased porosity. However, the drawback with larger pores is that the overall mechanical strength of the material is negatively impacted [39]. In the lyophilised form, bacterial cellulose has an elongation at break of approximately 5% and a typical tensile strength of 340 mPa, a Young’s modulus of 12 GPa, and a maximum strain order of 4%. However, wet bacterial cellulose has a maximum strain order of 20%, tensile strength of 400 mPa, and Young’s modulus of 130–145 GPa, indicating that wet bacterial cellulose has a greater level of elasticity [40,41,42].

Due to bacterial cellulose only comprising approximately 1% of the overall pellicle weight, with water (approximately 99%) comprising the remainder, it was also deduced that this material also has a significantly higher swelling ratio compared to commercially available cellulose [42]. It was further shown that the biomaterial is capable of regulating moisture content, which is ideal for applications in heavily exuding wounds [43]. This water-holding capacity of bacterial cellulose has been found to be 50 to 100 times its dry weight and is directly related to the overall surface area and distribution of pores within the material.

Another pertinent difference between commercially available cellulose derived from plants is that in bacterial cellulose, the crystallinity ratio is much higher (85–90%) in comparison to its commercial counterpart (60–65%). Moreover, in bacterial cellulose, there are no additional by-products that need to be removed, such as lignin and hemicellulose, allowing for a more streamlined and efficient production process [39].

All of these properties of bacterial cellulose have led to the successful commercialisation of bacterial cellulose hydrogels (e.g., Dermafill^®^ and Biofill^®^) for the treatment of burns, chronic ulcers, skin lesions, and periodontal disease [1]. Even though bacterial cellulose is not inherently antimicrobial itself, the unique 3D fibrillar network is highly porous and amenable to high loading with a controlled release of a range of antimicrobial agents, which can be delivered directly to the wound site [35,36].

The aim of this study was to evaluate the antifungal potential of biofunctionalised BC loaded with either thymoquinone, ocimene, and miramistin in comparison to the action of amphotericin B loaded in BC to determine whether alternative anti-fungal options can be used in the prevention and treatment of fungal wound infections.

## 2. Materials and Methods

### 2.1. Media and Reagents

Thymoquinone, ocimene, and amphotericin B, foetal bovine serum, glutamine, and antibiotics (penicillin G, 60 mg/L; streptomycin, 100 mg/L; standard amphotericin B, 50 μL/L) were purchased from Sigma-Aldrich (Irvine, UK). Miramistin was purchased from Carbosynth Ltd. (Compton, UK). Dimethyl sulfoxide (DMSO) reagent grade, sodium hydroxide, disodium phosphate, and citric acid were purchased from Sigma-Aldrich (Irvine, UK). Dextrose, bacteriological peptone, yeast extract, agar number 2, Sabouraud dextrose agar (SDA), malt extract agar (MEA), Hestrin and Schramm agar (HSA), Hestrin and Schramm media (HS), and Ringer’s solution were purchased from Lab M (Bury, UK). RPMI-1640 (Roswell Park Memorial Institute) was purchased from Fisher Scientific (Cramlington, UK). All media and reagents were prepared according to the manufacturer’s instructions.

### 2.2. Microorganisms

*Gluconacetobacter xylinus* ATCC^®^ 23770, Candida albicans ATCC^®^ 10,231 and Aspergillus niger ATCC^®^ 16,888 were obtained from the LGC Standards Ltd. (Middlesex, UK). Aspergillus fumigatus NCPF 2140 and Candida auris NCPF 8971 were obtained from Public Health England, Porton Down, UK. All organisms were obtained in lyophilised form and maintained at −20 °C before use. C. albicans, C. auris, A. fumigatus, and A. niger were revived on sterile SDA made to manufacturers’ specification, sterilised by autoclaving before use and incubated at 30 °C for 24 h to obtain maximum growth. G. xylinus was revived on sterile HSA at 37 °C for five days. Overnight cultures of G. xylinus were grown under agitation in HS broth medium at 37 °C for 24 h from stock plates to ensure the bacterial cells are well dispersed within the media, rather than conglomerating into a single bacterial pellicle.

### 2.3. Cell Culture

Human epithelial type 2 (HEp-2) cell lines were obtained from the American Type Culture Collection (ATCC^®^ CCL23™). The cells were cultured in RPMI-1640 containing 10% foetal bovine serum, 2 mM glutamine, and antibiotics (penicillin G, 60 mg/L; streptomycin, 100 mg/L; amphotericin B, 50 mg/L) under a humid atmosphere (37 °C, 5% CO_2_) for 96 h until confluent. Media were replaced every three days. The cells were washed with phosphate-buffered saline and passaged twice before use, again, once confluent.

### 2.4. Fungal Starter Cultures

Overnight cultures of fungi were prepared by using stock SDA plates to inoculate RPMI-1640 without bicarbonate, supplemented with 3-(N-morpholino)propane sulfonic acid (MOPS), 2% (*w*/*v*) glucose, and pH adjusted to 7 with 10 M NaOH. Then, the stocks were incubated statically at 30 °C for 24 h. C. albicans and C. auris pseudo-hyphae were produced using conditions published previously [44]. Briefly, overnight cultures of Candida species were collected by centrifugation at 1500 G for 15 min at 4 °C, washed twice with 0.15 M NaCl, resuspended in 0.15 M NaCl, and incubated at room temperature for 24 h to induce starvation. After 24 h of starvation, cells were inoculated into RPMI 1640 at a final concentration of 1 × 10^6^ cells/mL and incubated at 37 °C with shaking for 6 h.

### 2.5. Production and Purification of Bacterial Cellulose

BC production was carried out following the protocol reported in our previous paper [36]. Briefly, starter cultures of *G. xylinus* were used to inoculate HS media and incubated statically for 14 days at 37 °C. Following the incubation period, bacterial cellulose (BC) pellicles that formed as a raft on the surface of the HS media were aseptically harvested. The unpurified BC was initially heated to 100 °C in distilled water, which was followed by the addition of 2% (*w/v*) sodium hydroxide and reheating to 100 °C in fresh distilled water for a further hour or until the BC became fully transparent. Then, each pellicle was frozen at −20 °C before lyophilisation.

### 2.6. Preparation of Working Solutions and Loading of Antifungal Agents

Stock solutions, working solutions, and dilution series’ were conducted according to ISO 16256:2012(en) [45]. Briefly, 12,800 mg/L thymoquinone, ocimene, or miramistin and 3200 mg/L amphotericin B were made by dissolving each compound in DMSO. A dilution series following Table 1 and Table 2 were conducted to obtain working solutions at 200-fold the final concentration. Then, a 1:100 dilution was conducted to produce drug concentrations at twice the final concentration. The final concentration of amphotericin B ranged from 0.03 to 16 mg/L, and thymoquinone, ocimene, and miramistin ranged from 0.125 to 64 mg/L. Clean lyophilised BC pellicles were aseptically cut into 4 mm (used in cytotoxicity assays to fit 96-well plates) and 8 mm disks using a biopsy punch, which was then submerged in the varying concentrations of either thymoquinone, ocimene, miramistin, or amphotericin B and were placed on an orbital shaker (150 rpm) for 24 h in the dark at room temperature. Confirmation of loading was achieved through FTIR analysis

### 2.7. Minimum Inhibition Concentration (MIC)

The MIC_90_ (where the antifungal drug inhibits the growth of 90% of the organism) of thymoquinone, ocimene, miramistin, and amphotericin B was determined for all fungal strains using a standardised methods: CLSI M27 and M38 [46,47]. Briefly, RPMI without bicarbonate was supplemented with 3-(N-morpholino)propanesulfonic acid (MOPS), 2% (*w*/*v*) glucose and pH adjusted to 7 with 10 M NaOH. All fungal strains were grown on SDA, and suspensions were made in the RPMI-1640 to 0.5 McFarland standard at 530 nm, approximately 5 × 10^6^ CFU/mL. The microtiter plates were incubated for 24 h at 35 °C, and the OD570 was measured with a microtiter plate reader (SPECTROstar^®^ Nano, BMG Labtech, Ortenberg, Germany). All experiments were repeated in triplicate.

### 2.8. Minimum Fungicidal Concentration (MFC)

The MFC for all four antifungal compounds was determined by transferring 100 μL from all clear wells obtained during MIC assays (lack of turbidity is indicative of little impediment to growth) onto SDA plates and incubated overnight at 35 °C.

### 2.9. Quantification of Loaded Compound in BC

Lyophilised unloaded BC was aseptically cut into 8 mm disks and weighed. Then, the disks were individually placed into 1 mL aliquots of 50 mg/L (*w/v*) solutions of either thymoquinone, ocimene, miramistin, or amphotericin B for 24 h at room temperature under agitation (100 RPM). The concentration of 50 mg/L was chosen for all compounds, as it is twice the concentration of the highest MIC/MFC value and in accordance with standardised operating protocols [46,47]. After loading, the samples of BC were removed from the solutions and were again lyophilised by freezing at −20 °C for 24 h and lyophilised for a further 72 h and weighed again. All experiments were conducted ten times.

### 2.10. Zone of Inhibition (ZOI)

Disk diffusion assays were conducted using a modified Kirby–Bauer procedure [48] following internationally recognised standardised techniques in CLSI M44 and M51 protocols [49,50]. Briefly, Mueller–Hinton (2% (*w/v*) glucose) agar plates were flooded with overnight broth cultures (0.5 McFarland) of either *C. auris*, *C. albicans*, *A. fumigatus,* or *A. niger*. Control plates of Mueller–Hinton (2% glucose) agar flooded with filter sterile RPMI-1640. Once the plates were inoculated, four 8 mm BC disks which were loaded with varying concentrations of the antifungal drug, were placed onto the surface of the seeded plates in triplicate and were incubated for 24 h at 35 °C. Pure BC discs loaded with water were used as controls. After incubation, the diameter of the clear zone (ZOI), including the diameter of the 8 mm disk, was measured and statistically analysed using one-way ANOVA post hoc Tukey (*p* < 0.05) (GraphPad Prism V. 9.0.1(151), GraphPad Software, San Diego, CA, USA).

### 2.11. Cytotoxicity

The assay detects the reduction of MTT (3-(4,5-dimethylthiazolyl)-2,5-diphenyl-tetrazolium bromide) by mitochondrial dehydrogenase within the fungi to form blue formazan, which indicates that the mitochondria are functioning normally, and hence the measurement of cell viability. Briefly, 25 cells/μL HEp-2 in RPMI-1640 were added to each well in a 96-well plate and incubated for 24 h. After incubation, the cells were washed twice with phosphate-buffered saline (PBS), and fresh RPMI-1640 was added. Bacterial cellulose 4 mm disks, which had been loaded with 50 mg/L solutions of thymoquinone, ocimene, or miramistin, were then added and incubated for a further 24 h in triplicate (*n* = 4). Following incubation, MTT (0.5 mg/mL PBS) was added to each well and incubated at 37 °C for 3 h. The solubilisation of formazan crystals was achieved by mixing DMSO (100 μL/well), which was gently agitated for 10 min, and the absorbance was read at 570 nm using a microplate scanning spectrophotometer (SPECTROstar^®^ Nano, BMG Labtech). The following formula calculated toxicity level:

(1) $$Cytotoxicity\;\%=1-\frac{mean\;abs\;of\;antifungal}{mean\;abs\;of\;negative}\times 100\;Viability\;\%=100-cytotoxicity\;\%$$

To reduce test error level, MTT was added to wells without cells, and along with other wells, absorbance level was read and ultimately subtracted from the whole absorbance.

### 2.12. Scanning Electron Microscopy (SEM)

Bacterial cellulose was lyophilised for 24 h (Christ b 1,8-LSC plus, Martin Christ GmbH, Osterode am Harz, Germany) and then gold-coated (EM-Scope SC500). SEM analysis was undertaken on a Zeiss Evo 50 EP, SEM (Carl Zeiss AG, Oberkochen, Germany). The SEM was set to a 10 kV acceleration beam at 100 µA. The probe ampere was set at 36 pA, and the data were analysed on INCA software (version 7.2, ETAS, Stuttgart, Germany).

### 2.13. Fourier Transform Infrared Spectroscopy

Purified BC and BC loaded with the respective antifungal drugs were cut into 8 mm disks and again lyophilised and then analysed using a Bruker ATR FTRI spectroscope after running background scans (Bruker, Billerica, MA, USA). Each loaded BC sample was analysed in triplicate for each respective drug and was scanned 12 times at 400–4000 cm^−1^ at room temperature.

### 2.14. Statistical Analysis

All experiments in this project were conducted in triplicate, and all data presented are means± standard deviation (SD). Data recorded during all experiments were analysed statistically by one-way ANOVA post hoc Tukey (GraphPad Prism V. 9.0.1(151), GraphPad Software, San Diego, CA, USA) with (*p* < 0.05) for ZOI, MIC and MFC. *p* values for cytotoxicity studies were (*p* < 0.01).

## 3. Results and Discussion

The discovery of multi drug-resistant fungal pathogens increases the risk of medical complications whilst simultaneously limiting therapeutic options. The presented work is focused on the design and development of novel antifungal hydrogels for improvement of therapeutic efficacy of antifungal drugs such as thymoquinone, ocimene, and miramistin and compared to conventional drugs such as amphotericin B.

### 3.1. Preparation of BC Hydrogels and Characterisation

**Figure 3:**
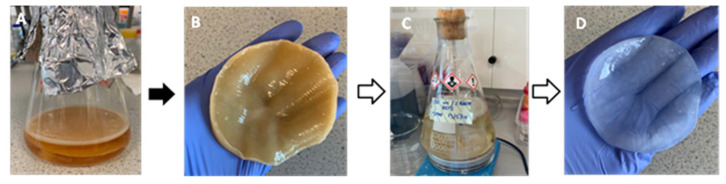
Biosynthesis of bacterial cellulose in Hestrin and Schramm medium, including pre- and post-purification. (**A**) static biosynthesis of BC which can be seen floating on top of the medium, (**B**) unpurified BC post harvest, (**C**) purification of BC in 2% (*w/v*) sodium hydroxide and, (**D**) purified BC.

The successful biosynthesis of bacterial cellulose is shown in Figure 3. The BC pellicles were harvested from the top of the growth media (A) and are opaque in colour (B), following purification by boiling in water and 2% (*w/v*) sodium hydroxide and again in water (C), then, the pellicles were removed and have become transparent after the purification process (D) as all remanence of growth media and *G. xylinus* cells were removed.

**Figure 4:**
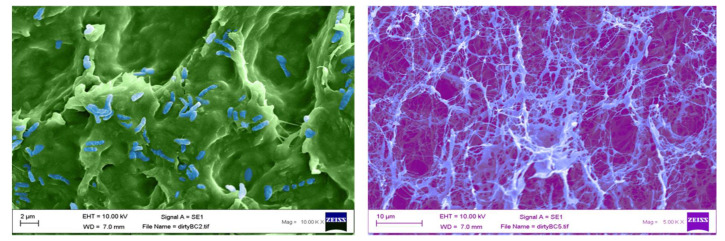
False coloured scanning electron micrographs of (**A**) unpurified (*G. xylinus* coloured blue), and (**B**) purified bacterial cellulose (cellulose nanofibres highlighted in light blue).

After biosynthesis, both unpurified and purified BC were lyophilised for 96 h and analysed using a scanning electron microscope. Figure 4A shows an unpurified sample of BC, which has been false coloured to highlight the closed matrix as it is laden with growth media and biomass, the bacteria, *G. xylinus*, can be seen highlighted in blue. Figure 4B shows an SEM micrograph of purified BC, and the nanofibrous matrix is now visible (blue) with typical voids (purple) also present.

**Figure 5:**
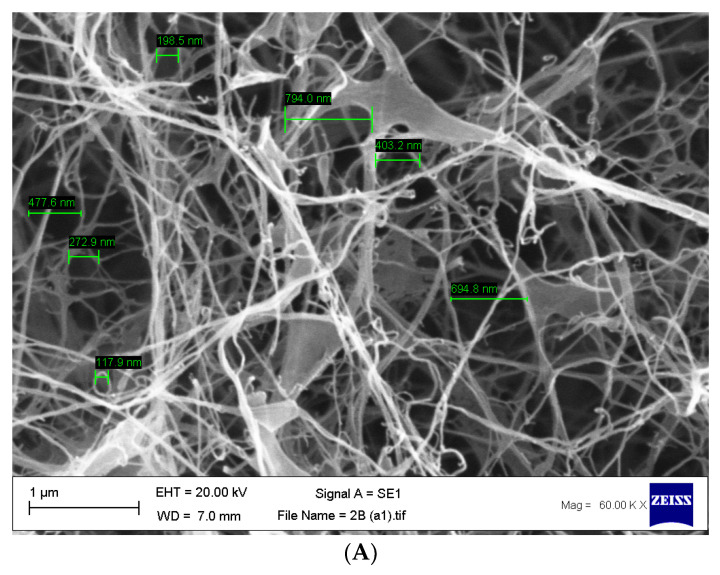

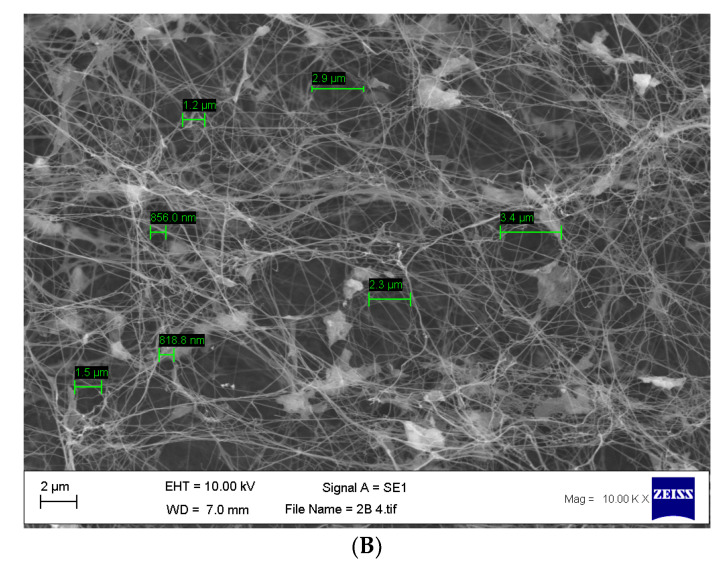
(**A**,**B**) Scanning electron micrographs of purified bacterial cellulose highlighting the size of the pores found within the matrix with an average range of 117.9 nm to 3.4 µm. *n* = 14, error bar 1 µm.

Once the bacterial cellulose was purified and lyophilised, SEM analysis was undertaken to confirm the pore size. Our results are concordant with previously published data [51], which show an average pore size of 117.9 nm to 3.4 µm (Figure 5A,B).

**Figure 6:**
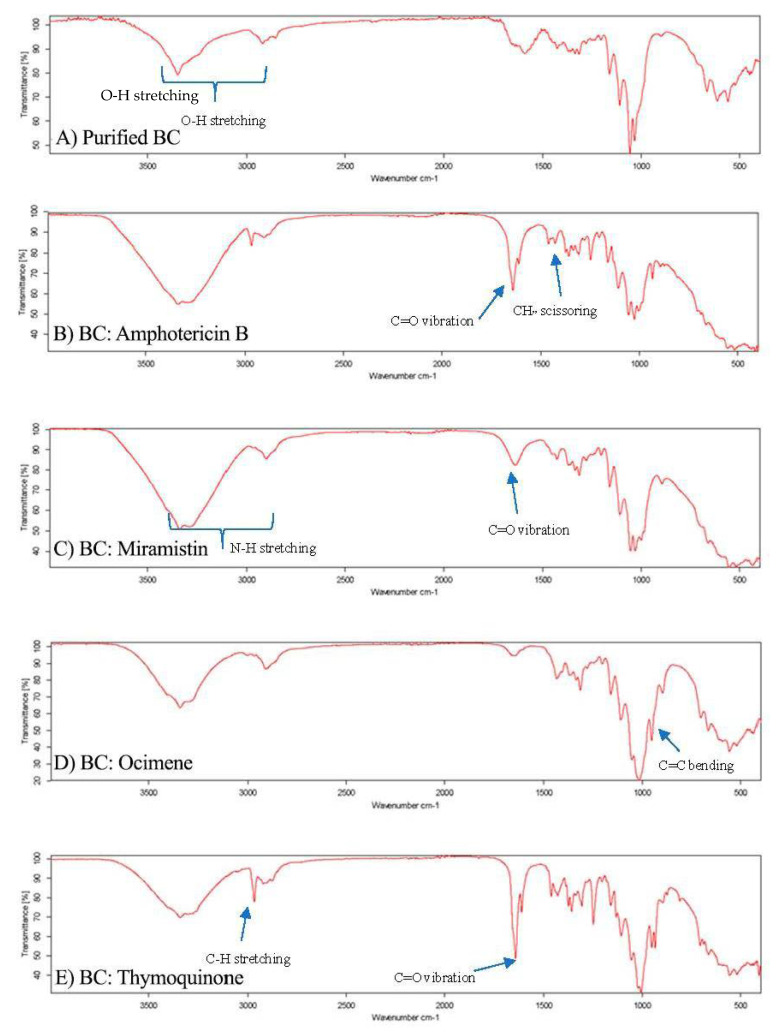
FTIR spectrographs of (**A**) purified BC, (**B**) BC: Amphotericin B, (**C**) BC: Miramistin, (**D**) BC: Ocimene, and (**E**) BC: Thymoquinone. (*n* = 3, 16 scans).

Following the characterisation of the purified BC via SEM, the lyophilised BC was aseptically cut using biopsy punches into 4 mm disks to be used in cytotoxicity assays and 8 mm disks used in disk diffusion assays. After this, the BC disks were placed into solutions of amphotericin b, thymoquinone, ocimene, or miramistin and were agitated overnight to ensure maximum loading of the antifungal agent. To confirm the successful loading, FTIR was performed on each of the samples (Figure 6A–F).

Each sample displayed characteristic O-H stretching between 2500 and 3300 cm^−1^ due to hydroxyl groups found in the bacterial cellulose matrix (Figure 6A). It is worth noting that all samples analysed displayed similar characteristic wavelengths to pure cellulose of 3330, 2894, 1614, 1370, 1159, and 1056 cm^−1^, as seen in research previously conducted [52]; however, subtle additions to these wavelength peaks in the fingerprint region were used to confirm the presence of the antifungal agents in the respective samples.

The FTIR spectrum for BC: amphotericin B (Figure 6B) shows three principal vibrations of the amphotericin B molecule: the band with a maximum at 1730 cm^−1^, which can be assigned to the vibration of C=O in the –COOH group, the CH_2_ scissoring vibrations (the band centred at 1460 cm^−1^), and the C=C stretching vibration, which is represented by peaks between 1486 and 1631 cm^−1^. Additionally, the peaks in the region of 3300–3400 cm^−1^ indicate stretching vibrations of the O–H group and the N–H stretching in concordance with previous research [53]. The appearance of peaks between 3400 and 3300 cm^−1^ in the FTIR spectra, Figure 5B, can be also attributed as a band of OH groups of BC involved in forming hydrogen bonds with the antifungal agent, indicating an interaction between BC and amphotericin B.

The FTIR spectrum for BC: miramistin (Figure 6C) shows two principal vibrations of the miramistin molecule: the signals from the amide group (NH–CO) were observed in the range of 3400–3200 cm^−1^ and C=O in the range 1650–1700 cm^−1^. In addition, peaks seen at 1650–1580 cm^−1^ suggest bending in N-H groups and stretching vibrations of C=C in the phenolic ring [54]. Moreover, the broad band seen between 3400 and 3300 cm^−1^ in Figure 6C, can also indicate on the formation of the hydrogen bonding between functional amide groups NHCO of miramistin and BC.

A strong peak observed at 3000–2900 cm^−1^ in Figure 6D corresponds to C–H stretching in the alkyne of ocimene. Peaks at 1605 cm^−1^ and 1640 cm^−1^ suggest C=C bonding. However, the confirmation of successful loading of ocimene was achieved by identifying a sharp peak at 892 cm^−1^ which corresponds to C=C bending in the vinylidene group, which is similar to other research [55].

The results of FTIR spectra for thymoquinone-loaded BC (Figure 6E) have assigned the existence of a variety of sharp, strong, and weak peaks as well as crucial functional groups that correspond to C=O, C-H, –CH_2_, –CH_3_, C=C, and C–O, suggesting the successful loading of thymoquinone within the BC sample. The intense band present at 2967 cm^−1^ corresponds to the C-H stretching of aliphatic groups, while the band observed at a higher wavenumber ≈3040 cm^−1^ was assigned to the stretching observed in the vinylic C–H in the C=C–H groups, which had previously been reported [56]. Additionally, the characteristic strong band of the carbonyl groups of a cyclohexadiene ring is observed at the wavenumber ≈1650 cm^−1^. As a result of FTIR analysis, we can confirm that the bacterial cellulose was successfully loaded with each antifungal drug, respectively.

### 3.2. Antifungal Studies

After successfully loading the drug compounds, we proceeded to conduct MIC_90_ and MFC studies to determine the optimum concentration to apply in disk diffusion and cytotoxicity assays. All MIC_90_ results (Table 3) obtained in this study are consistent with published MIC_90_ breakpoints where available in CLSI M60 and M61 standardised protocols [57,58]. This suggests that the test compounds have similar antifungal properties to amphotericin B, but on average, they are 69% less cytotoxic while maintaining similar antifungal profiles. This could prove beneficial in treating resistant and difficult to treat fungal infections, as compounds such as thymoquinone or ocimene, which appear to have similar antifungal profiles to amphotericin B, could be given without the harsh side effects that are often observed when administering amphotericin B.

In previous research by Abdel et al. (2013), they demonstrated that topical thymoquinone cream could be used safely against vaginal candidiasis in mice at concentrations of 10% (*w/v*) [59], which is twice the concentration used in this present study (Table 3).

**Figure 7:**
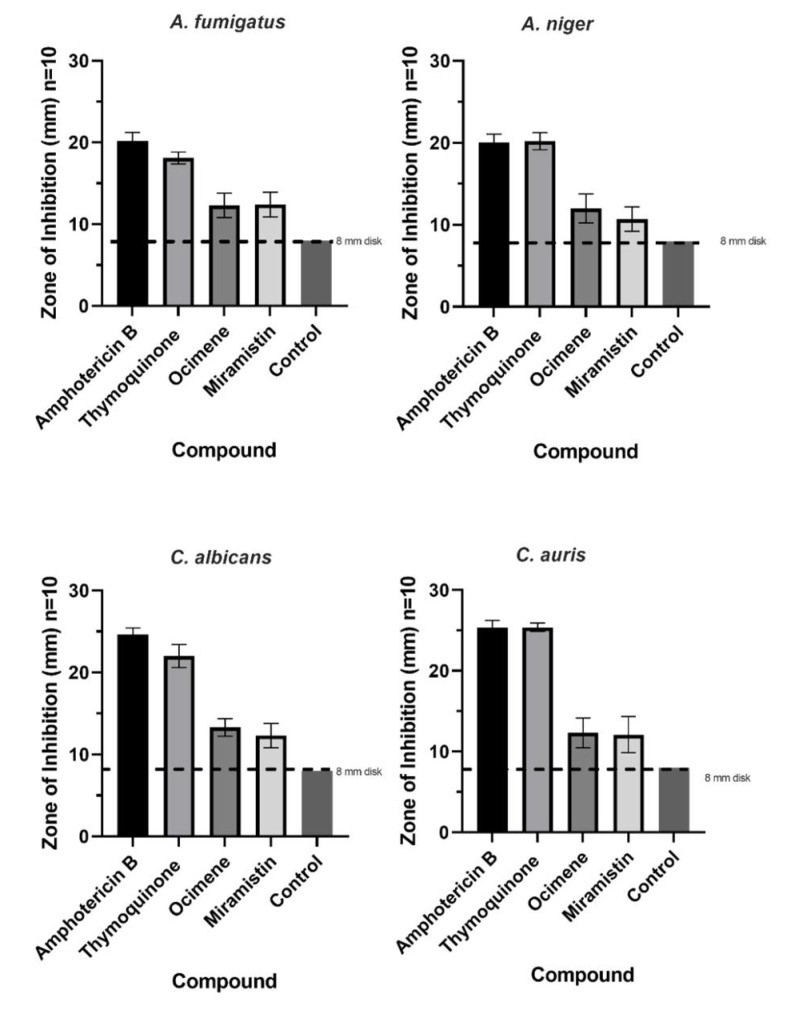
Mean zone of inhibition graphs for all four antifungal agents at a concentration of 50 mg/L loaded into 8 mm disks of purified BC tested against *A. fumigatus*, *A. niger, C. albicans,* and *C. auris*. Disks of pure BC were used as controls. Each test was conducted in triplicate 10 times (*p* ≤ 0.05, *n* = 10, error bars = SD).

**Figure 8:**
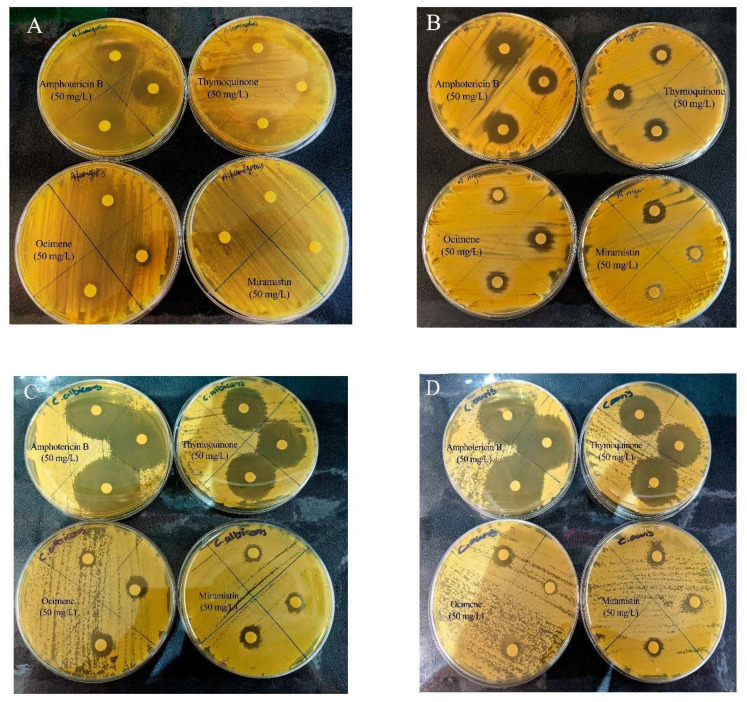
Representative disk diffusion assay plates showing the ZOI of bacterial cellulose disks loaded with amphotericin B, thymoquinone, ocimene, and miramistin against (**A**) *A. fumigatus*, (**B**) *A. niger*, (**C**) *C. albicans*, and (**D**) *C. auris* (*n* = 3).

The mean zones of inhibition for each compound at 50 mg/L, which is twice the concentration required as advised by CLSI for all fungi, are shown in Figure 7, and examples of these zones are shown in Figure 8. The disk diffusion assay for antifungal activity indicated that thymoquinone-loaded BC has comparable inhibitory effects against all four genera of fungi to amphotericin B with no significant difference (*p* > 0.05). However, ocimene and miramistin-loaded BC compared to amphotericin B against both *Candida* and *Aspergillus* species showed a significant difference (*p* < 0.05) in activity. Amphotericin B had a mean ZOI against all four genera of fungi of 22.53 ± 0.969 mm, while thymoquinone similarly had a mean ZOI of 21.425 ± 0.925 mm. However, the mean ZOI for ocimene and miramistin were 12.475 ± 1.536 mm and 11.875 ± 1.682 mm, respectively, as seen in Figure 8A–D.

Quantification of the drugs entrapped throughout the bacterial cellulose matrices were achieved by comparing the difference in weight of lyophilised unloaded BC to lyophilised loaded BC (Table 4). The difference in weight is directly proportional to the available free compound within the solution at a concentration of 50 mg/L (twice the required concentration to maintain consistency between compound concentrations), along with the ability for the BC to absorb up to 99% its weight in liquid, thus absorbing up to 99% of solubilised compounds available. Table 4 shows that all compounds were successfully loaded and retained within the BC matrix up to 78.95 ± 17.5%. Similarly processed disks were subsequently used in MTT assays to confirm any cytotoxic effects they may exert on HEp-2 cells.

The results from our study for amphotericin B and thymoquinone against both *Candida* species are similar to previous studies conducted; however, in these studies, the drugs were modified to be either liposomal or nanoparticulate, which may have impacted the overall MIC/MFC concentrations [60,61,62]. However, it should be noted that in the mentioned research, only Randhawa et al. (2015) [61] conducted their studies using internationally recognised protocols; therefore, the studies conducted by Khan et al. (2018) and Cavaleiro et al. (2015) [62,63] would benefit from being repeated using appropriate standardised protocols. Additionally, the results obtained for the antifungal activity of thymoquinone against all four genera of fungi in our study are similar to results published by Khader et al. (2009) [64]. They also showed that the MIC/MFC for yeasts ranged from 1.25 to 0.08 μg/mL and for non-dermatophyte fungi ≥10^−5^ μg/mL [64]. Their results may be slightly lower than our MIC/MFC data, as the study mentioned above used clinical isolates, which could show reduced resistance to the compounds.

The results show evidence that bacterial cellulose loaded with thymoquinone, ocimene, or miramistin display antifungal activity against different species of *Candida* and *Aspergillus*. This is especially poignant as both *Candida* species and *Aspergillus* species can develop resistance to commonly used antifungal drugs [5].

### 3.3. Cytotoxicity Studies

**Figure 9:**
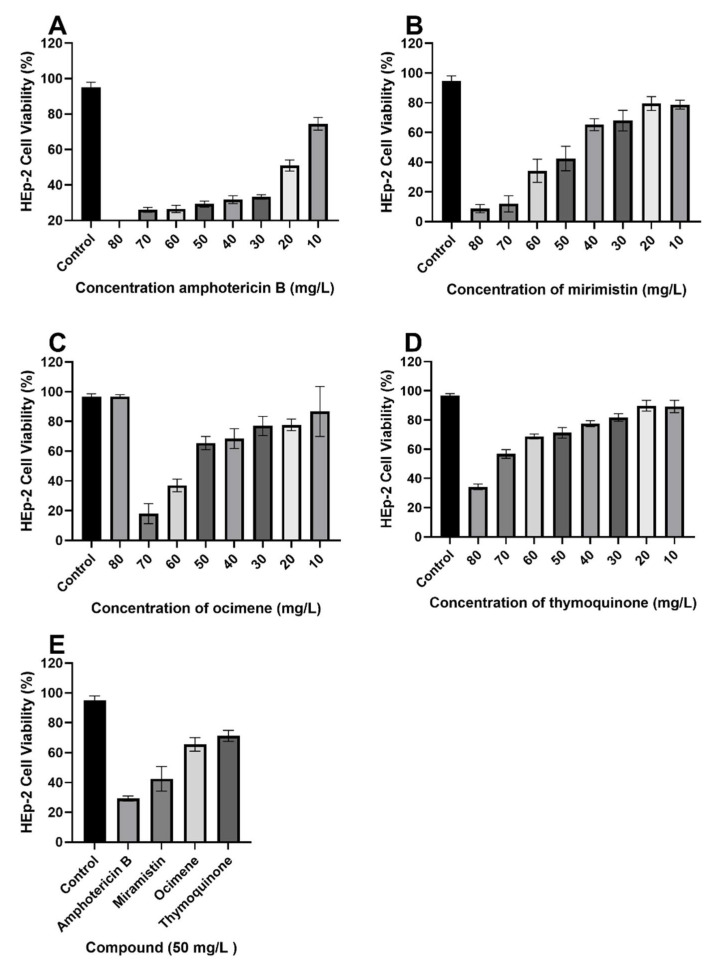
MTT cytotoxicity assay with varying concentrations of each respective drug, (**A**) amphotericin B, (**B**) miramistin, (**C**) ocimene, and (**D**) thymoquinone, against HEp-2 cells, (**E**) condenses data of 50 mg/L drug concentration from each graph (**A**–**D**) for ease of comparison. All tests were carried out in triplicate. Control was DMSO and RPMI-1640 (50:50) (*p* ≤ 0.01, *n* = 10, error bars = SD).

Once minimum fungicidal concentrations were determined, we proceeded to conduct cytotoxicity studies with concentrations of each respective drug ranging from 80 to 10 mg/L encompassing all values (Figure 9A–E) to find the most potent concentration while maintaining an acceptable level of toxicity toward HEp-2 cells. It is also shown through the cytotoxicity assays that all three test compounds have significantly lower (*p* < 0.01) cytotoxic effects against HEp-2 cells in comparison to amphotericin B; cell viability for 50 mg/L solutions of amphotericin B was 29.25 ± 1.708%, whereas for thymoquinone, ocimene, and miramistin, it was 71.25 ± 3.594%, 65.5 ± 4.435%, and 42.5 ± 8.266%, respectively.

**Figure 10:**
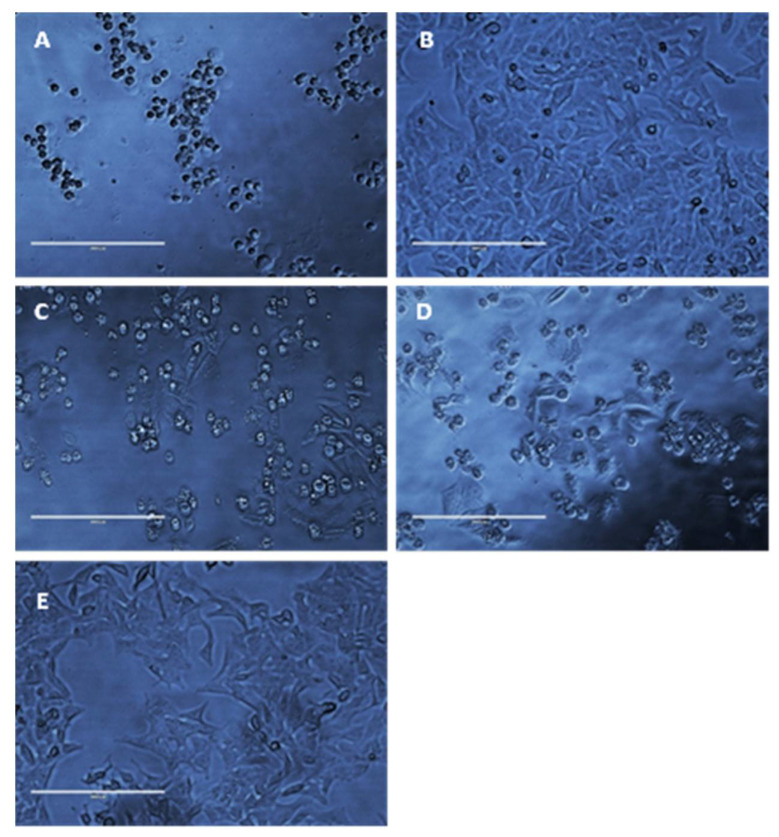
Representative photomicrographs of HEp-2 cells (40× magnification) after exposure to 50 mg/L amphotericin B (**A**), thymoquinone (**B**), miramistin (**C**), ocimene (**D**), and control (**E**) for 24 h. Control (**E**) was cultured without any addition of antifungal agents for 24 h in RPMI-40 (scale bar = 200 μm).

As a result of MIC/MFC assays, it was determined that a concentration of 50 mg/L (*w/v*) would be used in disk diffusion assays owing to an average cell viability rate of Hep-2 cells of 40% to 60% and because standard operating protocols advise using twice the drug concentration of the highest MIC. It is also worth noting that Figure 9E compiles cell viability rates for all drugs at a concentration of 50 mg/L (*w/v*); a significant difference (*p* < 0.05) can be seen in the survivability of HEp-2 cells when treated with thymoquinone, ocimene, and miramistin in comparison to amphotericin B, which showed a mean cell survival rate of 29.25 ± 0.854%. In contrast, thymoquinone at a concentration of 50 mg/L (*w/v*) showed a mean survival rate of 71.25 ± 1.797%. As shown by the 8 mm BC disk compound quantification assay, we can anticipate that up to 78.95 ± 17.5% of the free compound in solution will also be absorbed by the 4 mm BC disks used for the cytotoxicity assay. These data are supported by Khader et al. (2009), who concluded that ≥20 uM concentration of thymoquinone in vitro caused 6.37 ± 0.75% necrosis in hepatocytes [64]. The in vitro toxicity of miramistin was also supported by Osmanov et al. (2020) [65], who concluded that there were no cytotoxic effects seen at a concentration of 1000 mg/L against McCoy mammalian cell lines. Subsequent confocal microscopy of the HEp-2 cells after being exposed to the antifungal agents for 24 h can be seen in Figure 10A–E. The typical morphology (triangular) of HEp-2 cells can be observed in Figure 10E (control) with all previous images (Figure 10A–D) showing treated cells.

Figure 10A shows cells treated with amphotericin B, and as expected, they became detached from the base of the well and died, as evidenced by the circular appearance rather than being triangular in nature. Figure 9B–D show HEp-2 cells treated with thymoquinone, miramistin, and ocimene, respectively, and both show a positive correlation to the survivability data in Figure 9A–E. The vast majority of cells have retained their triangular appearance and have remained attached to the well’s base, suggesting cellular survival, which is in accordance with Uribe et al. (2013), who described the cytotoxic effects of amphotericin B in myofibroblast cell lines [66].

Additionally, it would be interesting to investigate the antifungal agents in vivo against animal models, which would elucidate the antifungal agents’ real potential as the hemocompatibility, along with biocompatibility of the overall biofunctionalised material system, could be collated. Moreover, future studies could be performed using clinical isolates to reduce the risk of selection bias. Secondly, further investigation into the mechanism of action of miramistin, ocimene, and thymoquinone would allow for a greater understanding of how these drugs exert their antifungal properties. Researchers have reported that thymoquinone and ocimene potentiate the mode of action of antibiotic compounds; however, research toward ocimene in this area is still lacking [67,68,69]. Nevertheless, as the studies conducted by Goyal et al. (2017), Liu et al. (2019), and Sarah et al. (2019) [67,68,69] were outside the remit of internationally recognised protocols, their results should be reconfirmed utilising appropriate standard protocols such as those produced by CLSI. Still, the test compound results for the antifungal potential in our study show promise and merit further investigation.

## 4. Conclusions and Future Prospects

The development of novel antifungal treatment options is an ever-pressing issue as the rise in the incidence of wound infections among both civil and military traumatic injuries, along with burns, trauma, and ulcers, are becoming more frequent. These injuries and subsequent infections by fungal pathogens are linked with increased mortality, risk of limb loss, prolonged stays in hospital, failure of the treatment regimen, and further systemic infection. As shown in this study, thymoquinone, ocimene, or miramistin-loaded bacterial cellulose wound dressings are promising antifungal hydrogels that could address the aforementioned issues. As shown through the MFC and cytotoxicity assays, these materials could be potentially used in the groups mentioned above owing to the high level of antifungal activity and low cytotoxic effects when compared to conventional antifungal drugs that could exasperate pre-existing conditions, namely amphotericin B [70].

The results of this study indicate that the implementation of thymoquinone, ocimene, or miramistin hydrogel wound dressings may depreciate the need to use other systemic antifungal compounds in the treatment of superficial and deep tissue wounds, which is also in line with currently antimicrobial stewardship agendas and has been developed to specifically target and reduce the incidence of antimicrobial resistance [71]. Prolonged states of trauma are usually associated with inappropriate therapy, which is well documented in being a causative factor toward the development of antimicrobial resistance [72,73]. The issue of antifungal resistance and the need to develop newer and more efficacious products was highlighted by The World Health Organisation in 2020 during the first meeting of the WHO antifungal expert group tasked with identifying priority fungal pathogens and subsequent treatment options [6].

Through this study, the preliminary antifungal properties of thymoquinone, ocimene and miramistin have been conducted against four commonly encountered fungi, which are causative agents in wound infections. The agents have been shown through internationally recognised disk diffusion and broth dilution assay protocols to possess a similar antifungal profile compared to amphotericin B. However, the marked difference is found within our agents’ cytotoxicity profiles, which are significantly lower (*p* < 0.01) than that of amphotericin B.

Future perspectives of this study will focus on the loading capabilities of bacterial cellulose and combinations of potential antifungal agents to increase the bioactivity to a broader spectrum of organisms. Additionally, as the water-holding capability of the material is so high, this introduces issues of adherence of the wound dressing to the skin. Further investigations are required to develop a method by which the biomaterial can be modified to become adherent, possibly taking advantage of the ionic pores (Figure 5) within the cellulose matrices. Additionally, further investigations into the time release of each compound from BC will be conducted with appropriate time kill studies to determine how long these materials remain bioactive.

Following SEM characterisation of purified BC, it is clear that the pore sizes found within the matrices are generally uniform in size, around 117.9 nm to 3.4 µm, which is in concordance with existing research [38]. It is also postulated that there are two forms of pore that can be seen. The main pores, which are easily observed, fall within the mentioned dimensions; however, there are also indications that there are more superficial pores that are much larger in the region of 200 µm in diameter. This development in identifying various sizes and conformations of pores would allow future developments to be pursued in the area of incorporating additional compounds with a much larger structure, such as bioactivated zeolites loaded with antimicrobial metal ions. Table 3 highlights the efficacy of bacterial cellulose to absorb a large amount of free compound within a solution, showing up to 78.95 ± 17.5% successful loading and retainment throughout the entire material via physical absorption. This is agreeable to published data for the swelling ratio of bacterial cellulose [36].

The data collected during this study have highlighted the efficacy and high in vitro tolerability of our agents in use as an antifungal and would benefit greatly from further investigation. As we have highlighted in this study, the importance of utilising naturally occurring products is paramount in fighting the burdening issue of antifungal resistance [74,75]. It is clear that pharmaceutical agents such as amphotericin B are usually accompanied by severe side effects that make their use intolerable to individuals while having low efficacy to their target organism [76,77]. To the best of our knowledge, we are the first to successfully load thymoquinone into bacterial cellulose hydrogels, thus producing a novel wound dressing. We have also determined that thymoquinone and amphotericin B have similar antifungal potencies; however, the thymoquinone’s overall cytotoxicity is significantly lower (*p* < 0.01).

## Figures and Tables

**Table 1:** Scheme for preparing amphotericin B in DMSO with a final concentration of 0.06 to 16 mg/L.

Step	Concentration (mg/L)	Source	Volume of Antifungal (μL)	Volume of Solvent (μL)	Intermediate Concentration (mg/L)	Concentration (mg/L) after 1:100 Dilution with Double Strength RPMI 2% (*w/v*)
1	3200	Stock	200	0	3200	32
2	3200	Stock	100	100	1600	16
3	3200	Stock	50	150	800	8
4	3200	Stock	50	350	400	4
5	400	Step 4	100	100	200	2
6	400	Step 4	50	150	10	1
7	400	Step 4	50	350	50	0.5
8	50	Step 7	100	100	25	0.25
9	50	Step 7	50	150	12.5	0.125
10	50	Step 7	25	175	6.25	0.06

**Table 2:** Scheme for preparing thymoquinone, ocimene, and miramistin in DMSO 0.26 to 64 mg/L.

Step	Concentration (mg/L)	Source	Volume of Antifungal (μL)	Volume of Solvent (μL)	Intermediate Concentration (mg/L)	Concentration (mg/L) after 1:100 Dilution with Double Strength RPMI 2% (*w*/*v*)
1	12,800	Stock	200	0	12,800	128
2	12,800	Stock	100	100	6400	64
3	12,800	Stock	50	150	3200	32
4	12,800	Stock	50	350	1600	16
5	1600	Step 4	100	100	800	8
6	1600	Step 4	50	150	400	4
7	1600	Step 4	50	350	200	2
8	200	Step 7	100	100	100	1
9	200	Step 7	50	150	50	0.5
10	200	Step 7	25	175	25	0.26

**Table 3:** MIC and MFC of antifungal agents against fungal isolates.

Fungi	Amphotericin B		Thymoquinone		Ocimene		Miramistin	
	MIC_90_ (mg/L)	MFC (mg/L)	MIC_90_ (mg/L))	MFC (mg/L)	MIC_90_ (mg/L)	MFC (mg/L)	MIC_90_ (mg/L)	MFC (mg/L)
*A. fumigatus*	0.06–1	0.07–1	1.3–2	1.5–2	0.7–1	1.6–2	21	27
*A. niger*	0.06–1	0.09–1	0.9–2	1–2	0.4–0.9	1–2	18-20	25
*C. albicans*	0.03	0.06–1	4.9–5.2	6.2–7	0.3–0.9	0.3–0.9	1.3–3	2.6–4
*C. auris*	0.05	0.08–1	7.9–8.2	8.4–9.7	1.2–1.9	2–2.5	1.4–2.8	3–5

**Table 4:** Quantification of antifungal compounds loaded within lyophilised 8 mm disks of bacterial cellulose (*n* = 10, ±SD).

Drug	Unloaded BC (mg)	Loaded BC (mg)	Average Compound Loaded (µg)
Thymoquinone	1.6375 ± 0.364	1.678 ± 0.357	42.0 ± 8
Ocimene	1.5500 ± 0.208	1.588 ± 0.207	37.4 ± 9
Miramistin	1.5750 ± 0.175	1.615 ± 0.183	46.0 ± 8
Amphotericin B	1.7000 ± 0.221	1.733 ± 0.220	32.5 ± 10
Control	1.6200 ± 0.125	1.627 ± 0.120	7.00 ± 2

## Data Availability

The data presented in this study are openly available.
